# Trilateral retinoblastoma: neuroimaging characteristics and value of routine brain screening on admission

**DOI:** 10.1007/s11060-012-0922-4

**Published:** 2012-07-18

**Authors:** Firazia Rodjan, Pim de Graaf, Hervé J. Brisse, Sophia Göricke, Philippe Maeder, Paolo Galluzzi, Isabelle Aerts, Claire Alapetite, Laurence Desjardins, Regina Wieland, Maja Beck Popovic, Manuel Diezi, Francis L. Munier, Theodora Hadjistilianou, Dirk L. Knol, Annette C. Moll, Jonas A. Castelijns

**Affiliations:** 1 Department of Radiology, VU University Medical Center, Postbox 7057, 1007 MB Amsterdam, The Netherlands; 2 Department of Radiology, Institut Curie, 75248 Paris, France; 3 Department of Diagnostic and Interventional Radiology and Neuroradiology, University Hospital, 45122 Essen, Germany; 4 Department of Radiology, University Hospital, 1011 Lausanne, Switzerland; 5 Department of Neuroimaging and Neurointerventional (NINT), Azienda Ospedaliera e Universitaria Santa Maria alle Scotte, 53100 Siena, Italy; 6 Department of Pediatric oncology, Institut Curie, 75248 Paris, France; 7 Department of Radiotherapy, Institut Curie, 75248 Paris, France; 8 Department of Ophtalmology Surgery, Institut Curie, 75248 Paris, France; 9 Department of Pediatric Oncology, University Hospital, 45122 Essen, Germany; 10 Department of Pediatric Hematology-Oncology Unit, University Hospital, 1011 Lausanne, Switzerland; 11 Jules Gonin Eye Hospital, University Hospital, 1011 Lausanne, Switzerland; 12 Department of Ophtalmology, Azienda Ospedaliera e Universitaria Santa Maria alle Scotte, 51300 Siena, Italy; 13 Department of Epidemiology and Biostatistics, VU University Medical Center, 1181 HV Amsterdam, The Netherlands; 14 Department of Ophtalmology, VU University Medical Center, 1181 HV Amsterdam, The Netherlands

**Keywords:** Trilateral retinoblastoma, Pineoblastoma, MR imaging, Pediatric oncology, Head and neck

## Abstract

Trilateral retinoblastoma (TRb) is a rare disease associating intraocular retinoblastoma with intracranial primitive neuroectodermal tumor. Treatment is difficult and prognosis is poor. This multicenter study evaluates clinical findings and MR imaging characteristics of associated intracranial tumors in Rb patients. Clinical data of 17 patients (16 TRb and 1 quadrilateral Rb patients) included time intervals between Rb and TRb diagnosis and presence of baseline brain-imaging (BBI). Two reviewers reviewed all images individually and one reviewer per center evaluated their images. Consensus was reached during a joint scoring session. Studies were reviewed for tumor location, size and imaging characteristics (signal intensity (SI) on T1- and T2-weighted images, enhancement pattern and cystic appearance). Of 18 intracranial tumors, 78 % were located in the pineal gland and 22 % suprasellar. All tumors showed well-defined borders with mostly heterogenous enhancement (72 %) and isointense SI on T1- (78 %) and T2-weighted images (72 %) compared to gray matter. The majority of pineal TRbs showed a cystic component (57 %). TRb detected synchronously with the intraocular tumors on BBI (*n* = 7) were significantly smaller (*P* = 0.02), and mainly asymptomatic than TRb detected later on (*n* = 10). Overall, 5-year-survival of TRb patients detected on BBI was 67 % (95 % CI 29–100 %) compared to 11 % (95 % CI 0–32 %) for the group with delayed diagnosis. TRb mainly develops in the pineal gland and frequently presents with a cystic appearance that could be misinterpreted as benign pineal cysts. Routine BBI in all newly diagnosed Rb patients can detect TRb at a subclinical stage.

## Introduction

Trilateral retinoblastoma (TRb) is a disease associating unilateral or bilateral retinoblastoma (Rb) with an intracranial midline primitive neuroectodermal tumor (PNET) which usually arises in the pineal gland (PG) (77 %) [1]. In hereditary Rb patients, the neural ectoderm destined to form both retinal and pineal tissue is prone to develop multifocal neoplasms. This results in histological similar but separate located tumors [2]. The risk of developing TRb in Rb patients is less than 0.5 % for sporadic unilateral disease [3], 5–13 % in sporadic bilateral disease, and 5–15 % in familial bilateral Rb [1]. Patients with TRb frequently present with signs of intracranial hypertension [3–7]. Few long-term survivors are reported, and, especially in symptomatic patients, prognosis is poor [1, 3, 8–10].

Previous studies on TRb detection, neuroimaging screening, and prognosis all focused on time intervals between detection of Rb and TRb (metachronous tumor development) [1, 10–13]. Reported median time between Rb and TRb diagnosis is 21 months [1, 3, 9, 10]. However, Kivela et al. [1] reported tha,t with inclusion of brain MR screening during first MRI examination for Rb (i.e., baseline brain imaging; BBI), approximately 50 % of TRb cases can potentially be found. These are considered synchronous tumors, detected on baseline MRI. Approximately another 25 % of TRbs can be found during the first year after Rb detection. However, recent literature states that TRb is rarely present at diagnosis of Rb [14]. We hypothesize that the exact prevalence of synchronous occurrence of TRb and Rb in literature is underestimated. In most studies, it remains unclear whether BBI was performed at Rb diagnosis, at some time-point during follow-up or only in a later stage for detection of symptomatic TRb; and if imaging was performed with CT or MR. This complicates the evaluation of “true” synchronous TRb in literature.

Only few radiological articles on TRb have been reported, and these were mainly individual case reports. To our knowledge, only two studies described radiologic findings on MRI in trilateral retinoblastoma, both within small groups of patients [10, 15]. Because of these modest study populations, it is relevant to identify specific MRI characteristics of TRb in a larger group of patients.

The primary purpose of this multicenter study was to evaluate clinical findings and MRI characteristics of associated intracranial tumors in Rb patients. The secondary purpose was to assess clinical, radiological and prognostic differences between TRb depicted on BBI and those depicted later on.

## Materials and methods

### Patient population

This retrospective study was performed in agreement with the recommendations of the local ethics committees within a European Retinoblastoma Imaging Center (ERIC) with five participating Rb centers. Review of clinical records between 1991 and 2010, revealed 17 Rb patients with MRI and intracranial tumors. TRb was diagnosed on the basis of histopathological confirmation [surgery or presence of tumor cells in cerebrospinal fluid (CSF)] or clinical disease progression during follow-up MRI. TRb was defined as a mass lesion in the PG or suprasellar region in Rb patients. Tumor in both PG and suprasellar regions in combination with bilateral Rb was classified as a quadrilateral Rb (QRb).

### Record review

Clinical records were reviewed for tumor laterality, family history for Rb, age of Rb diagnosis, time interval from Rb to TRb diagnosis and TRb diagnosis to death or last follow-up date. Symptoms at first presentation of TRb and treatment received for Rb and TRb were recorded. Laboratory records were analyzed for tumor^ ^cells in CSF acquired by lumbar puncture (LP) performed either at diagnosis or during follow-up. Particular attention was paid to the presence of BBI, which is necessary to evaluate the simultaneous occurrence of TRb at Rb diagnosis. TRbs were categorized in synchronous or metachronous tumors to the intraocular tumor. Patients with bilateral retinoblastoma, a positive family history of retinoblastoma or mutations in the RB1 gene found in chromosomal/DNA analysis were classified as hereditary. Disease progression was defined as either tumor recurrence, intracranial or intraspinal leptomeningeal spread or distant metastases.

### Image review

Patients underwent various imaging protocols for the assessment of TRb. MRI sequences varied in different institutions. Brain MRI protocols at least included either sagittal or transverse unenhanced T1-weighted images or T2-weighted images in 14 patients. Post-contrast T1-weighted images of TRb were available in 16 patients.

Two observers (JC and PdG) with, respectively, 22 years and 10 years experience individually reviewed all MRI examinations and one radiologist from each participating center (HJB, PG, PM and SG) evaluated their images. Agreement was reached during a joint scoring session. MR images were evaluated for mass lesions in the PG and suprasellar regions and for leptomeningeal tumor dissemination. Regarding the TRb, maximal axial diameter (MAD) at diagnosis, tumor border, presence of tumor necrosis, tumor aspect (solid, solid with cystic component,; or complete cystic), SI on T1- and T2-weighted images compared to gray matter, aspect of contrast enhancement, presence of vessel encasement, and hydrocephalus and leptomeningeal metastases were scored.

### Statistics

Statistical calculations were performed using SPSS v.15.0 (SPSS, Chicago, IL, USA). BBI and MAD were analyzed by using the Mann–Whitney test. Difference in mean MAD between pineoblastomas and suprasellar tumors was analyzed using an independent *t* test. Associations between other clinical dichotomous parameters and BBI were assessed using Fisher exact tests. A 95 % CI for 5-year survival was calculated based upon the Kaplan–Meier survival function. A *P* value of less than 0.05 was considered statistically significant.

## Results

### Clinical findings

Clinical data of part of this study have been previously reported (Table 1) [1]. Ten patients had familial Rb (59 %) and 11 patients also a positive RB1-gene mutation (65 %). Sixteen patients (94 %) were classified as hereditary Rb. Mean age of Rb diagnosis was 9 months (median age 5 months) and of TRb 26 months (median age, 23 months). Mean time-interval between detection of Rb and TRb was 18 months (median, 14 months). In none of the patients, TRb was found before Rb.

**Table 1:** Clinical patient characteristics

Patient (Rb lat)	Confirmation TRb	Age Rb	Age TRb	Int. Rb–TRb	Year Rb date	BBI	Death	Int TRb death	Int TRb FU	Treatment RB (OD; OS)	Treatment TRb
1 (B)	Histopathology	3	52	49	1986	No	Yes	57	57	PT	ChT, EBRT
2 (B)	CSF	2	39	37	1990	No	Yes	21	21	EBRT	ChT, EBRT
3 (B)	Histopathology	5	10	5	1991	No	Yes	7	7	En; EBRT	Palliation
4 (B)	DP on MR	23	38	15	1992	No	Yes	0	0	EBRT	Palliation
5 (B)	CSF	2	26	24	1992	No	Yes	13	13	CrT; En.	ChT
6 (B)	DP on MR	12	12	0	1997	Yes	Yes	14	14	CrT, En.	No
7 (U)	CSF	3	57	54	1997	No	No		93	No; En.	ChT
8 (B)	Histopathology	2	42	40	1998	No	Yes^a^	11	11	ChT	ChT, EBRT, surgery
9 (B)	DP on MR	3	3	0	2000	Yes	No		74	ChT/CrT; En.	ChT
10 (B)	CSF	17	17	0	2001	Yes	Yes	7	7	ChT	ChT
11 (B)	CSF	3	15	12	2001	No	NA^b^		3	CrT; En.	ChT
12 (B)	CSF	7	23	16	2002	No	Yes	16	16	En; En	ChT
13 (B)	CSF	1	31	30	2002	No	Yes	15	15	ChT; CrT	ChT, surgery
14 (B)	CSF	10	10	0	2003	Yes	No		50	ChT	ChT
15 (B)	CSF	12	12	0	2005	Yes	No		63	ChT, CrT;	ChT
16 (U)	Histopathology	10	10	0	2006	Yes	No		56	ChT	ChT, surgery
17 (U)	No	38	38	0	2008	Yes	NA	NA	NA	No; En.	NA

*Rb lat* laterality Rb, *B* bilateral Rb, *U* unilateral Rb, *Int. Rb–TRb* interval between Rb and TRb in months, *Int TRb death* interval TRb and death in months, *Int TRb FU *interval of follow-up in months,* CSF* cerebrospinal fluid, *DP* disease progression, *BBI* baseline brain imaging, *PT* plaque therapy, *ChT* chemotherapy, *EBRT* external beam radiation therapy, *CrT* cryotherapy, *NA* not available

^a^Due to intoxicity after chemotherapy

^b^Lost to follow-up after 3 months with progressive disease

Nine patients had signs of intracranial hypertension, whereas the other 8 patients were asymptomatic at detection. LP at baseline were performed in 7 patients (positive for tumor cells in 5 patients) and during follow-up in 11 patients (positive in 5 additional patients). Histopathologic specimens were available in 4 TRbs and were classified as PNETs. Rb was treated with external beam radiotherapy (EBRT) (mean age 10 months, median 5 months) in 3 out of 10 metachronous patients. These patients developed TRb after a mean interval of 19 months (range 5–37 months). Rb was treated with chemotherapy in 2 out of 10 metachronous TRb patients (mean interval 35 months; range 30–40 months). Treatment for TRb was initiated in 13 patients. Two patients received palliative treatment because of tumor spread, one patient was not treated because of parental refusal, and one patient was lost of follow-up.

### MRI characteristics of TRb

MRI characteristics are summarized in Table 2. In our group of 17 patients, 18 intracranial tumors were detected.

**Table 2:** Imaging characteristics

Patient	Intracranial tumor location	MAD	Tumor border	AL	Necrosis	SIT1	SIT2	Enhancement	LM	Hydrocephalus	VE
1	PG	25	Well defined	Solid	No	NA	NA	Homogeneous	No	Yes	Yes
2	PG	55	Well defined	PC	Yes	Isointense	Hypointense	Heterogeneous	No	Yes	Yes
3	PG	59	Well defined	PC	Yes	Isointense	Isointense	Heterogeneous	No	Yes	Yes
4	PG	49	Well defined	PC	Yes	Hypointense	Isointense	Heterogeneous	Yes	Yes	Yes
5	PG	18	Well defined	Solid	No	Isointense	Hypointense	Heterogeneous	Yes	Yes	No
6	PG	9	Well defined	Cystic	Yes	Isointense	Isointense	Heterogeneous	No	No	No
7	PG	51	Well defined	PC	Yes	Isointense	Isointense	Heterogeneous	Ntb	Yes	No
8	PG	22	Well defined	Solid	Yes	Isointense	Isointense	Heterogeneous	No	Yes	No
9	PG	13	Well defined	Cystic	No	NA	NA	Heterogeneous	No	No	No
10	SS	15	Well defined	Solid	No	Isointense	Isointense	Homogeneous	No	No	No
11	PG	13	Well defined	Solid	Yes	Isointense	Isointense	NA	Ntb	No	No
12	PG	11	Well defined	Solid	Yes	NA	Isointense	Heterogeneous	No	No	Yes
12	SS	44	Well defined	Solid	No	NA	Isointense	Homogeneous	No	No	Yes
13	PG	33	Well defined	Solid	Yes	Isointense	Isointense	Heterogeneous	Yes	Yes	No
14	SS	23	Well defined	Solid	No	Isointense	Isointense	Homogeneous	No	No	No
15	SS	34	Well defined	PC	Yes	Isointense	Isointense	Heterogeneous	Yes	No	No
16	PG	21	Well defined	Cystic	Yes	Isointense	Isointense	Heterogeneous	No	No	No
17	PG	11	Well defined	Cystic	Yes	Isointense	NA	Heterogeneous	No	No	No

*PG* pineal gland, *SS* supra sellar, *MAD* maximal axial diameter, *AL* aspect lesion, *PC* partly cystic, *NA* not available, *SI T1* signal intensity on T1-weighted images compared to gray matter, *SI T2* signal intensity on T2-weighted images compared to gray matter, *LM* leptomeningeal metasteses, *VE* vessel encasement

**Fig. 1:**
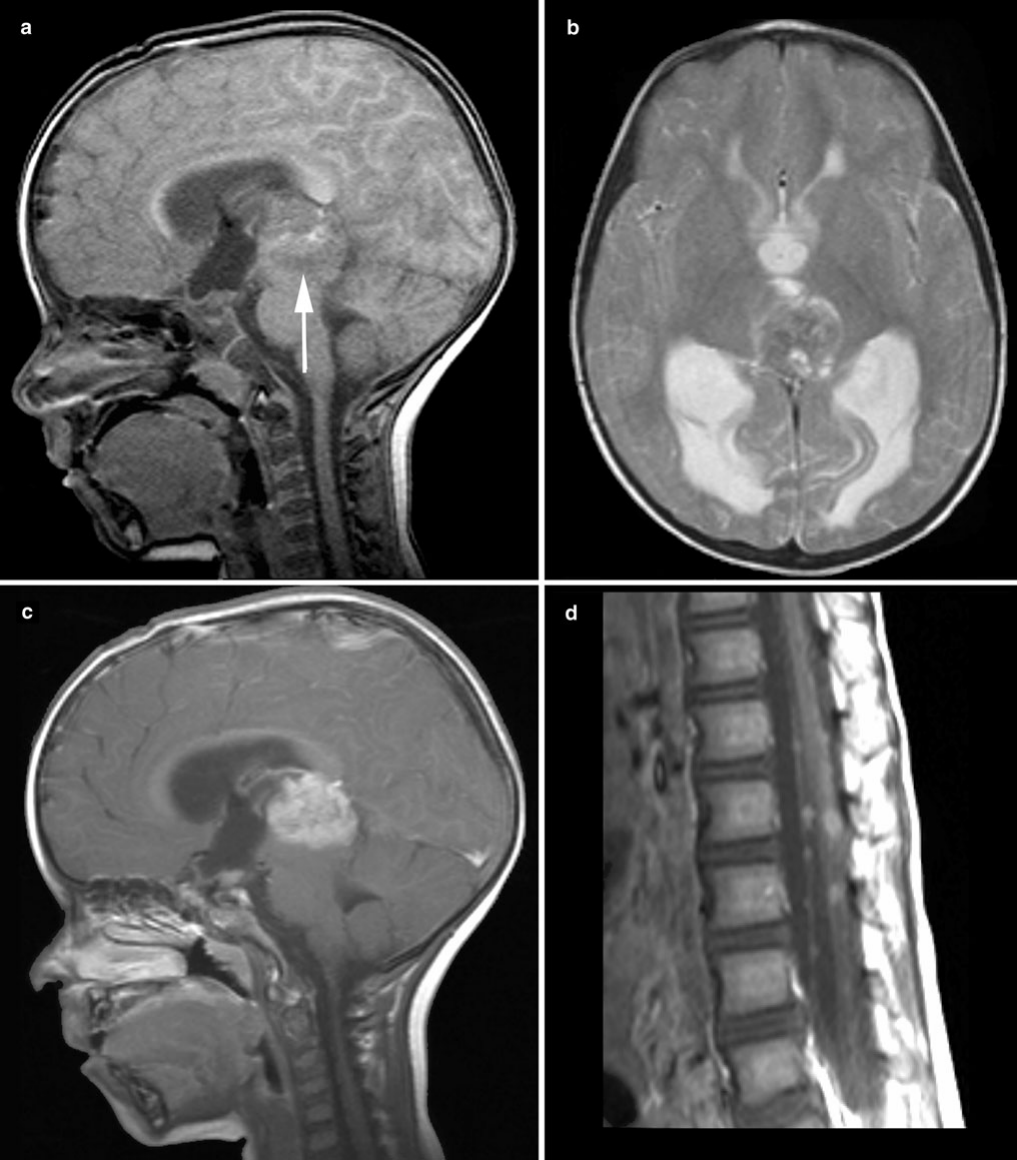
Solid pineoblastoma with hydrocephalus and extensive leptomeningeal metastases. Sagittal T1-weighted (**a**), axial T2-weighted (**b**), contrast-enhanced sagittal T1-weighted images of the brain (**c**) and spine (**d**) of patient 13. Pineoblastoma showed mostly isointense SI on both T1-weighted (**a**) and T2-weighted (**b**) MR images with respect to gray matter and homogenous contrast-enhancement (**c**). The large tumor mass (33 mm) showed compression on the brainstem (mesencephalon) and cerebral aquaduct (**a**, **c**) with secondary hydrocephalus (**b**). Multiple nodular leptomeningeal tumor seedings are present in the spinal canal (**d**)

**Fig. 2:**
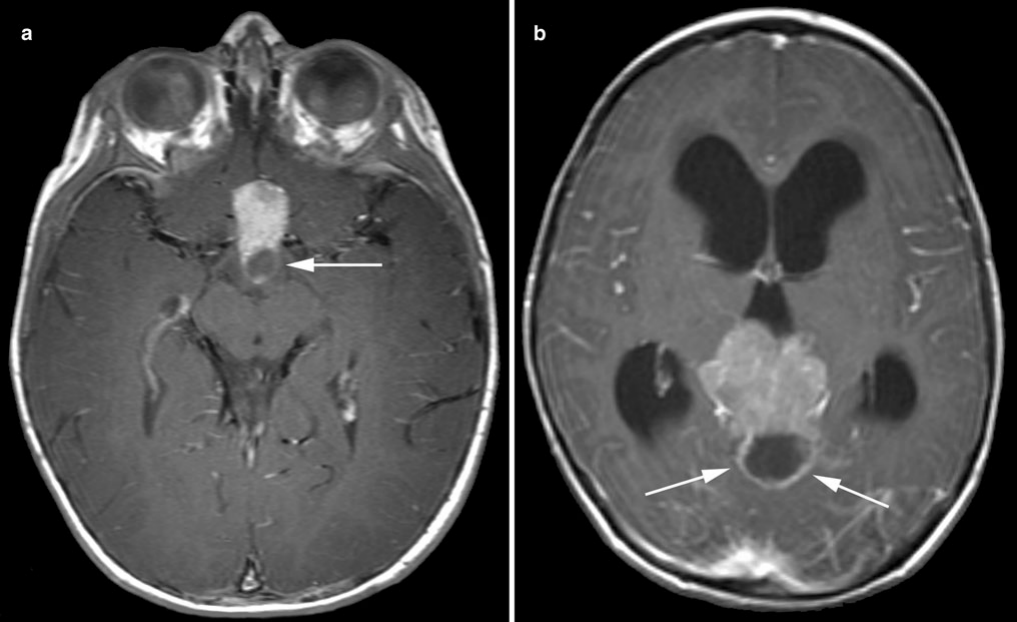
Suprasellar and pineal gland trilateral retinoblastoma. Contrast-enhanced axial T1-weighted images showing solid tumor masses with cystic components in both the suprasellar region (patient 15) (**a**) and pineal gland (patient 7) (**b**). The pineal gland mass causes a secondary hydrocephalus because of brainstem compression (**b**)

**Fig. 3:**
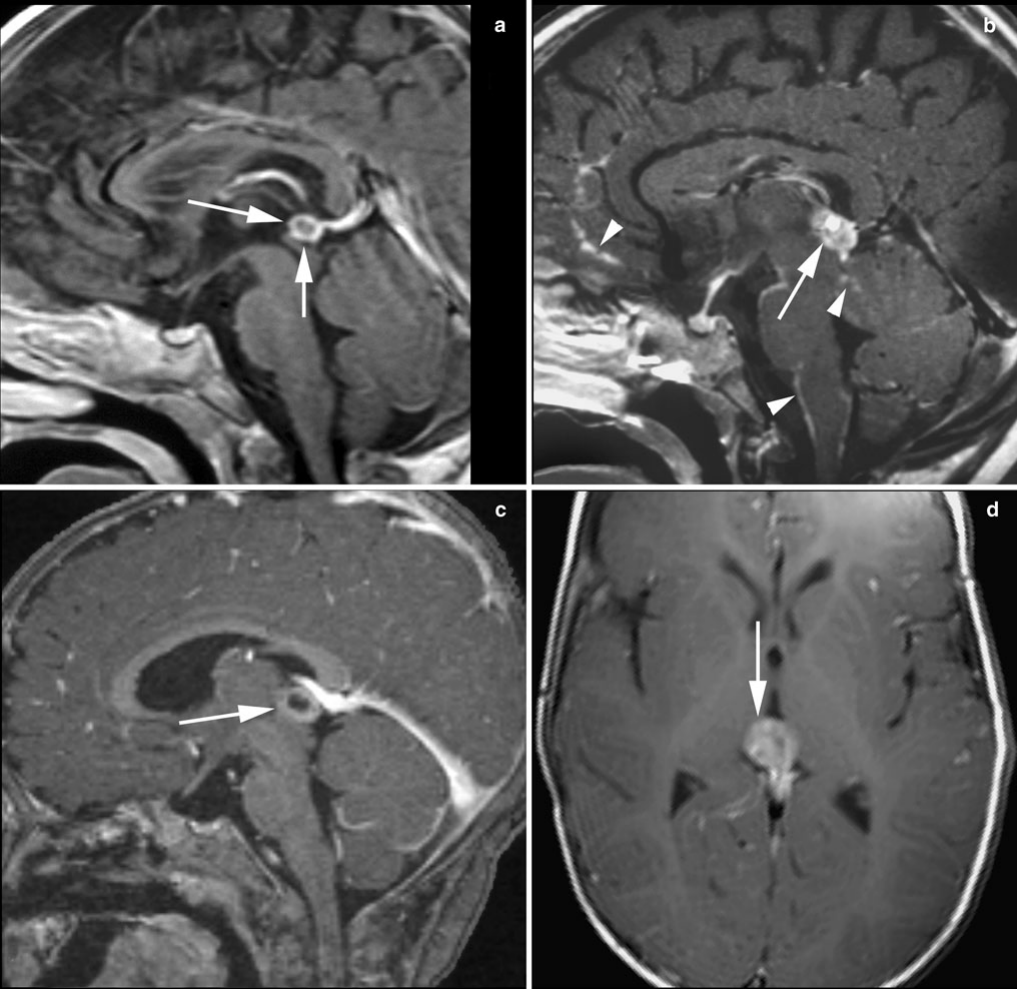
Pineoblastoma presenting as suspicious cyst. Contrast-enhanced sagittal (**a**–**c**) and axial (**d**) T1-weighted images of the brain in patient 6 (**a**, **b**) and patient 16 (**c**, **d**). The pineal gland in **a** shows an irregular cyst wall with tiny nodules, which progressed into a solid tumor with diffuse (nodular) leptomeningeal metastases 14 months later after treatment refusal (**b**). The pineal gland in patient 16 mimics a pineal cyst on the sagittal image (**c**), but shows a solid part of the lesion on the axial image (**d**), suspicious for pineoblastoma

Of the 14 pineoblastomas, 6 (42 %) showed a completely solid aspect (Fig. 1a), 4 (29 %) solid with cystic component (Fig. 2), and 4 (29 %) were completely cystic with an irregularly thickened rim (Fig. 3). Pineoblastomas mimicking pineal cysts showed an irregular (patient 6; Fig. 3a) or thickened (patient 16; Fig. 3c) cyst wall, sometimes with tiny nodules. Follow-up imaging in patient 6 showed progression of the pineal lesion into a solid tumor with diffuse leptomeningeal metastases 14 months after refusal of treatment (Fig. 3b). Patient 16 showed an obvious solid tumor part on the axial MR images (Fig. 3d). Secondary hydrocephalus occurred in 8 patients (57 %) with pineoblastoma (Figs. 1b, 2b) and leptomeningeal metastases in 3 patients (21 %) (Figs. 1d, 2b). One suprasellar tumor showed a homogenous solid aspect with a cystic component (Fig. 2a).

Overall, the mean MAD was 30 mm (range 9–59 mm).

### Clinical and radiological patterns according to time of diagnosis

BBI was available in 7 cases and in all Rb and TRb were diagnosed simultaneously (mean age 15 months, median 12 months; range 3–38 months). These 7 patients did not have any signs of intracranial hypertension at first presentation.

In the remaining 10 patients without BBI, mean interval between Rb and TRb diagnosis was 27 months (median 24 months; range 5–54 months). Symptoms of intracranial hypertension occurred in 8 patients. A significant difference was observed in tumorsize (*P* = 0.02) and hydrocephalus (*P* = 0.002) in favour of patients with BBI. In 1 patient with TRb, no symptoms occurred, and in 1 QRb patient, symptoms could not be retrieved from the clinical records. Furthermore, other differences were observed in the patients with BBI compared to patients without BBI. Tumor size was significantly smaller in patients with BBI [mean MAD 18 mm (range 9–34 mm)] compared to patients without BBI [mean MAD 35 mm (range 11–59 mm)] (*P* = 0.02). Hydrocephalus (*P* = 0.002) occurred more often in patients without BBI and thus in larger tumors. Lumbar puncture in patients with BBI was positive in 29 % of the cases and 70 % in patients without BBI (*P* = 0.15).

In addition, more synchronous tumors were detected after the year 2000 as illustrated in Table 1, because BBI was more routinely included in current imaging protocols.

### Survival

One out of 17 patients was lost to follow-up and excluded from survival analysis. One out of the remaining 16 patients was treated with chemotherapy and lost to follow-up after 3 months with progressive disease, and one died from intoxicity after intensive chemotherapy.

Five out of 16 patients (33 %) are still alive (mean survival, 67 months; median 63 months, range, 50–93 months). Four of these 5 patients (80 %) presented with synchronous TRb detected on BBI and were free of disease (mean survival, 61 months; range 50–74 months). The other survivor was diagnosed with a pineoblastoma of 51 mm (54 months after Rb diagnosis without BBI). This patient had local tumor recurrences but is still in second complete remission, 93 months after complete resection of the pineoblastoma and intensive chemotherapy.

The remaining 10 patients died after a mean interval of 24 months. Eight presented with metachronous TRb and without BBI. Cause of death in these 10 patients included local spread of the initial TRb (3 patients), leptomeningeal metastases (4 patients), tumor recurrence (2 patients), and side effects of intensive chemotherapy (1 patient).

Difference in survival of PNET early detected with BBI compared to those with delayed diagnosis was not significant (*P* = 0.064). The overall 5-year survival of PNET detected on BBI was 67 % (95 % CI 29–100 %) compared to 11 % (95 % CI 0–32 %) for the group without BBI.

## Discussion

The most important imaging finding of this retrospective analysis is that the majority of the pineoblastomas in our study were partially or totally cystic. Other main findings are that TRbs detected synchronously with the Rb on BBI were significantly smaller, more frequently asymptomatic, and could have a better prognosis compared to TRbs found after diagnosis of Rb (metachronous TRBs).

In the literature, the simultaneous occurrence of Rb and intracranial tumor is rare [14, 16]. Kivela et al. [1] reported that intracranial tumors were detected before Rb diagnosis in 3 % of the cases, 14 % simultaneously with Rb, and 83 % after Rb diagnosis. However, the incidence of synchronous TRb is probably underestimated, as historically little documentation about the presence of BBI at Rb diagnosis is available. Most TRbs described are diagnosed after first presentation with symptoms and signs of intracranial hypertension. Diagnosis and treatment for retinoblastoma is usually completed by then [3–7]. In our study, in all patients with synchronous tumors detected on BBI, significantly smaller TRbs were detected compared to metachronous TRb. Furthermore, 70 % of patients with metachronous tumors presented with symptoms due to intracranial hypertension and 80 % died due to their intracranial tumor. This indicates that the majority of metachronous tumors could have been detected in an earlier stage if BBI would have been performed. Remarkably, the majority of synchronous tumors were detected in TRb patients after the year 2000, as cerebral imaging was performed more frequently in our centers. We found a lower median time-interval of 14 months in our group compared to a median time interval of 21 months mentioned in literature [1, 13]. In these studies, however, the majority of the TRbs (83 and 62 %, respectively) were detected after diagnosis and treatment for Rb, while in our study a higher rate of synchronous tumors (41 %) were present.

Pineoblastomas and suprasellar tumors presented as typically well-defined lesions with relatively isointense SI on T1-weighted and T2-weighted images compared to gray matter. Contrast enhancement in these tumors was mostly heterogenous due to cystic components or tumor necrosis. Similar SIs on T1-weighted images were reported in 4 and 8 patients, respectively, on MRI [10, 15], but diverse enhancement patterns and SIs on T2-weighted images have been described [13, 15, 17, 18]. Hydrocephalus was a typical complication of large pineoblastomas. Therefore, we stress the need for BBI to detect smaller TRbs.

The majority of the pineoblastomas in our study were partially or totally cystic. Pineal cysts have been reported in Rb patients but not associated with hereditary Rb [19]. The presence of suspicious pineal cystic tumors, however, are a point of discussion [19–24]. Because of life-threatening side effects that may be related with curative aggressive treatment in TRb patients [25], it is important that cysts are not misinterpreted as tumor. Pineal cysts are diagnosed if (1) an enlarged PG is present, (2) with a hypointense central region with respect to white matter on T1-weighted-images and isointense with respect to CSF on T2-weighted images, and (3) a thin wall of 2 mm or less with discrete rim enhancement after gadolinium injection [20]. Although these criteria are formulated, pineal lesions in retinoblastoma are causing radiological dilemmas, especially if the cyst wall is irregularly thickened (>2 mm) or shows a fine nodular aspect of the wall [19, 20]. In our study, only 6 out of 14 pineoblastomas were completely solid, whereas 29 % (4 tumors) had both a cystic and solid component, and 4 tumors mimicked a pineal cyst. Hence, the need for imaging characteristics of early stage (cystic) pineoblastoma and follow-up scheme in suspicious cystic lesions of the PG is necessary to separate these from benign pineal cysts. Identification of such criteria is only possible in a large group of suspicious cystic PGs in Rb patients. Because these tumors are rare, a multicentric prospective study is necessary to define evident criteria for detection of early stage (cystic) pineoblastoma. Meanwhile, we recommend that pineal cystic lesions depicted on BBI should be classified into three groups: (1) “probably benign pineal cyst”, (2) “obvious cystic pineoblastoma”, or (3) “suspicious pineal cyst”. The first group contains patients with a cystic PG with discrete rim enhancement and a thin smooth wall; we recommend repeating MRI once after 6 months and, if stable, no further follow-up. The third group requires close MR follow-up after 3 months. As, currently, MRI of every new Rb patient is performed routinely in most centers, screening could easily be obtained by performing at least one brain MR sequence [26]. This screening could be achieved by a post-contrast 3D T1-weighted sequence with 1 mm slice thickness, and if a cystic portion is detected in the PG, an additional 2 mm T2-weighted sequence or thin slice 3D T2/CISS can further characterize the lesion.

In our series, a trend was observed for a better survival of patients who had PNET detected early with BBI. However, due to the small sample size, this difference did not reach significance. Several studies advise brain imaging screening in Rb patients in order to detect TRb in an early stage [1, 9, 27]. Although improvement of prognosis in TRb patients is important, caution with screening programs should be considered. First, prognosis of TRb patients detected by screening compared to patients with symptomatic disease should be evaluated. Duncan et al. [12] were the first to evaluate screening for TRb with CT at baseline and additional brain MRI every 6 months in 83 hereditary Rb patients. No improved outcome was observed despite early diagnosis. Kivela et al. [1], discovered that screening identified TRb in an earlier stage, but without better survival. This indicates that longer survival was due to lead-time bias. A disadvantage of early detection without better outcome is severe treatment-related morbidity and distress in these children leading to lower quality of life. In recent literature, however, high-dose chemotherapy has successfully been introduced for TRb, gradually leading to an increase in survival time [14, 28]. Especially, TRb detected in an early stage could benefit from these new treatment strategies, since reported survivors are almost inevitably the synchronous or early metachronous patients. These early metachronous patients (TRb diagnosed a few months after Rb diagnosis) should be classified as “missed synchronous” rather than “early metachronous”. Therefore, we stress the need for routine brain MRI in every single newly diagnosed retinoblastoma patient on admission, which is a potentially simple and (cost-)effective screening method for early TRb detection. The value of extending brain MRI screening after BBI is under discussion and therefore sporadically applied in European retinoblastoma referral centers.

The rare incidence of TRb in all participating Rb centers in Europe is in agreement with the observed declining incidence of TRb over the last decades [29–31] and is still a matter of debate. An increasing use of neoadjuvant chemotherapy for intraocular retinoblastoma (chemoreduction) preventing development of TRb has been suggested by Shields et al. [31], who registered fewer TRb since the introduction of chemoreduction as primary treatment for Rb. However, cases of TRb are reported even after an intensive scheme of chemoreduction therapy in advance [32]. In our study, 2 out of 10 metachronous TRb patients received chemotherapy and still developed TRb. The decreasing incidence of TRb could be due to the declining use of EBRT in patients with hereditary retinoblastoma [33]. In 3 patients, intraocular Rb was treated with EBRT, and these patients developed metachronous pineoblastomas.

A limitation of this study is the absence of BBI in all included metachronous TRbs. Therefore, the true incidence of metachronous TRb is still likely to be overestimated. Also, the small size of our patient cohort was a study limitation for statistical analysis.

In conclusion, TRb mainly develops in the PG and frequently presents with a cystic appearance that may be misleading. We recommend a three-group classification of pineal cystic lesions depicted in Rb patients. Routine BBI in all newly diagnosed Rb is strongly recommended as it may detect TRb in a subclinical and potentially curable stage.
